# 

**Published:** 1916-03

**Authors:** 


					﻿OUR NEW SUBSCRIBERS
Dr. Ii. S. McMullins____Tenn Dr. L. C. Adams ____________N C.
Dr. H. H. Workman ______8. C. Dr. J. W. Bone ------------Okla.
Dr. V. D.	Washburn________Del.	Dr.	C.	F. Ifawlev ____.Ariz.
Dr. C. W. Fulton_____LN. Mex. Dr. B. F. Clutter _________Texas
Dr. J. A.- Sparks _________Kv.	Dr.	H.	A. Reese--------Ariz.
Dr. S. G.	Zinke ___________Ky.	Dr.	W. H. Anderson_____Texas
Dr. J W.	Bastine_________Del.	Dr.	H.	B. Justice______Okla.
Dr. J. M.	Hampton ________Ark.	Dr..	W.	T. Gabbert ______'rk.
Dr. E. J. Stahl________AV. Va. Dr. R. N. Duffy___________N. C.
Dr. E. R. Fitch __________Ky. Dr. John W. Wright_________Okla.
Dr. J. H. Steenberger__W.	Va.	Dr.	R.	G. Milinder______Okla.
Dr. Paul C. Starkey____W.	Va,.	Dr.	L.	D. Latham ________Okla.
Dr. G. C. Robertson____W.	Va.	Dr.	Wm. H. Furman_______N. C.
Dr. F. F. Fadeley______N Mex.	Dr.	R.	T. Burt--------Tenn.
Dr. Geo. A. Bridge ______Ariz. Dr W. W. Harlam---------W. Va.
Dr. W. G. Bryan__________Ariz. Dr. S. W. Reynolds--------Okla.
Dr. J.	M.	King____________Ark.	Dr.	P.	N. Atkins --------Okla.
Dr. Chas. L. Smith_______Texas	Dr.	A.	L.	McInnis_______Okla.
Dr. II.	S.	Woodward_____Texas	Dr.	J.	A.	Jacoby-------Okla.
Dr. H.	G.	Camper ______  Texas	Dr.	H	P	Hurley ------Texas
Dr. H. M. Jarrell _____W. Va. Dr. A. A. Parker -----------Md.
Dr. O. G. Doughertv_______Ky. Dr. J. C. Lawson------------S. C.
Dr.	H. McLeod _______.___Al‘a.	Dr.	C. W. Birnie________S.	C.
Dr.	Win. Mann ___________N.	C.	Dr.	W. J. Crossland —---S. C.
Dr	Geo. E. Kornegay____.V	C.	Dr.	J A. Norton---------S.	C.
Dr.	O. C. Daniels________N.	C.	Dr.	J. 8. Jacoby---------Okla.
Dr. F. S. Robertson-----W. Va. H. A. Stroud-----------------Ark.
Dr. E. T. Kelly _______________ C. G. Willis_____________W. Va.
Dr. A. H. Gregg_________W. Va. Arthur Hooks ________________Va.
Dr. S. R. Lucas-----------S. C. J. D. Morris ________________Ga.
Dr. F. K. Rhodes ---------S. C. J. B. Thomas --------------Texas
Dr. F. C. Ferguson_______Texas Wade H. Brannon______________Ala.
Dr. H. M. Batson_______W. Va. F. W. Steiner__________________Md.
Dr. A	C.	Owings----------S.	C.	I. V. McGlasson__________Texas
Dr. C.	F.	Ross------------S.	C.	H. Wesley	Yeats__________Okla.
Dr. C.	L.	Hunsucker_______N.	C.	Harrv A.	Reese _________Ariz.
Dr. J.	L.	Anderson________S.	C.	McPherson	& Adams_______Texas
Dr. T.	G. Sharp-----------S.	C.	H.	W	Bass --------------Ala.
Dr. B.	A. Everett---------S.	C.	G.	D.	Vicks-------------N. C.
Dr. R.	H. McFaden---------S.	C.	F.	E.	Rushing___________Okla.
Dr. J	H. Hunter__________S.	C.	S.	W.	Rankin ____________N. C.
Dr. J. D. Harrison--------S. C. E. A. McPheeters____________Ariz.
Dr. V.	M. Long____________N.	C.	E.	B.	Dunlap ___________Okla.
Dr. Robt. Wriston ------W Va. Chas. Griffith_______________Tenn.
Dr.	J. G. Anderson------N.	C.	F.	M.	Sandiffer ________Tenn.
Dr.	S. W. Rankin _____N.	T.	W.	W. Porter_____________Tenn.
Dr.	J. L. Whitehead_____N.	C.	J.	W.	Fenn ______________Ala.
Dr.	H. C. McDowell______S.	C.	G.	D.	Lillard ____________Ky.
Dr. D. S. Porter__________S. C. R. E. McBride____________N. Mex.
Dr. P. K. Swintzer--------S. C. W. S. Southerland _________Texas
Dr. R. W. Timberlake W. Va. A. L. Mclnnes__________________Okla.
Dr. E. H. McClendon ________Ga. L. H. Forman ____________W. Va.
Dr. S. A. Hardman_______W. Va. J. S. Jacoby________________Okla.
Dr. W. P. Herbert_________N. C. L W. Humphreys___________W. Va.
Dr. A. B. Nelson -------W.	Va.	W. R. Pierce ____________Dela.
Dr. H. F. Lawson___________W	Va.	P. W. Atkins ____________Okla.
Drs. Bradford & Sharpe----S. C. C. K. Kercheval______________Ky.
Dr. R. W. Fisher_________W.	Va.	F. S. Robertson ________W.	Va.
Dr. S. P. Shirkey________W.	Va.	John Whitehead____________N. C.
Dr. W. N. Haynes_________W.	Va.	K. M. Jarrell __________W.	Va.
Dr. .1. R. Youmans ---------Ga.	Cochran & Smith _________Tenn.
Dr. D. L. Yost _________W.	Va.	J. E. Rader_____________W.	Va.
Dr. J. E. Mahoney----------Okla.	Jno. F. Bradley___________Ark.
Dr. B. D. Tyler -----------Kv.	J. F. Son________________Okla.
Dr. R.	L.	Cockfield-------S.	C.	L.	P.	Molloy _____________Ky.
Dr. Walter Wright_________Okla. H. A. Mood ________________S. C.
Dr. F.	L.	Carpenter ------S.	C.	M.	H.	Cravens___________Texas
Dr. G. T. Grisinger_____W. Va. A. F. Wicks_________________Miss.
Dr. S.	M.	Browne----------S.	C.	J.	H.	Marshall _________Texas
Dr. H. C. Martin________W. Va. Wm. T. Potter_______________N. C.
Dr. F. B. Morgan --------Texas E. R. Gordon _______________Miss.
Dr.	R.	H. Bryant__________N.	C.	J.	A. Rawlings __________Texas
Dr. H. C. Slaughter_____W. Va. J. P. Scales_________________Ala.
Dr.	C.	S. Sherrell _______N.	C.	J.	T. Reddick _____________Ky.
Dr.	C.	S. Howell_________S.	C.	C.	R. Berry _____________Miss.
Dr.	B.	M. Montgomery_____S.	C.	A.	B Leach_______________Texas
Dr.	I.	A. Macon _________S.	C.	FL G. Walcott ___________Texas
J. D Phillips ___________Texae W. H. Black___________________La.
J. W. Riley _____________Okla. L. T. Waller ______________Texas
H. R. Giles _____________Texas Edith Gerhold ______________N. C.
				

